# Case report: Lymphocytic-plasmacytic and eosinophilic enterocolitis presented with marked eosinophilia and basophilia in a cat

**DOI:** 10.3389/fvets.2023.1153702

**Published:** 2023-09-04

**Authors:** Jin-Young Kim, Tae-Sung Hwang, Dong-In Jung, Kun-Ho Song, Joong-Hyun Song

**Affiliations:** ^1^Department of Veterinary Internal Medicine, College of Veterinary Medicine, Chungnam National University, Daejeon, Republic of Korea; ^2^Institute of Animal Medicine, College of Veterinary Medicine, Gyeongsang National University, Jinju, Republic of Korea

**Keywords:** Basophilia, cat, eosinophilic gastroenteritis, inflammatory bowel disease, lymphocytic-plasmacytic enterocolitis

## Abstract

Inflammatory bowel disease is a common condition in cats, characterized by recurring gastrointestinal signs with histologic evidence of intestinal inflammation. A 9-month-old neutered male Sphynx cat was presented with a 5-week history of vomiting and hematochezia. Conservative patient management with a therapeutic gastrointestinal formula, antibiotics, and antiemetics resulted in a positive response to treatment, with relapse of signs when the medications were discontinued. A new finding of marked eosinophilia and basophilia was identified 3 months after the initial presentation. Colonoscopy revealed cecal erosions and a surgical biopsy with histopathology confirmed a diagnosis of lymphocytic-plasmacytic and eosinophilic enterocolitis. For this diagnosis, the patient was treated with prednisolone, tylosin, and metronidazole. Antibiotics were gradually tapered as the cat showed clinical improvement. The patient showed resolution of the gastrointestinal signs, and the numbers of eosinophils and basophils returned within the reference range 8 weeks after the treatment began. Basophilia and eosinophilia has been reported in conjunction with feline T-cell lymphoma. However, marked basophilia accompanying eosinophilia is extremely rare in cats with inflammatory bowel disease. We herein provide clinical details, including ultrasonography, endoscopy, histopathology, and disease course of feline lymphocytic-plasmacytic and eosinophilic enterocolitis with marked basophilia and eosinophilia. This case highlights the importance of considering enteritis as potential diagnoses when eosinophilia and basophilia are concurrently observed in cats.

## 1. Introduction

Basophils and eosinophils are important blood leukocytes. There are many cytokines associated with eosinophil and basophil production and proliferation, including interleukin-5 (IL-5), interleukin-3 (IL-3), and granulocyte-macrophage-stimulation factor (GM-CSF) (1). Among them, IL-5 and IL-3 are cytokines most closely related to the production of eosinophils and basophils, respectively. Recent studies also revealed that basophils and eosinophils could induce each other (1). IL-4 derived from basophils upregulates vascular cell adhesion molecule 1 (VCAM-1) in endothelial cells, inducing eosinophils to infiltrate the tissue. When eosinophils are activated, IL-3 and IL-5 are secreted, by which basophils are induced. However, even though eosinophils and basophils interact with each other at sites of inflammation, basophilia accompanying eosinophilia is extremely rare in cats. Most reported cases are associated with feline alimentary T-cell lymphoma (2).

Inflammatory bowel disease (IBD) is a collective term that describes recurrent gastrointestinal signs with histologic evidence of intestinal inflammation (3). IBD can be classified as lymphocytic-plasmacytic enterocolitis (LPE), eosinophilic enteritis (EE), granulomatous enteritis, and neutrophilic enteritis by its histological characteristics, with LPE being the most frequent histologic form in cats (4). In cats, since the clinical signs of IBD, such as vomiting, diarrhea, weight loss, and loss of appetite, are similar to those of low-grade intestinal T-cell lymphoma (LGITL), histopathology is required for the definitive diagnosis (5–7).

In this report, we describe basophilia accompanying eosinophilia in a cat, which has rarely been reported in lymphocytic-plasmacytic and eosinophilic enterocolitis. We herein describe clinical details and the disease course of feline lymphocytic-plasmacytic and eosinophilic enterocolitis with marked basophilia and eosinophilia.

## 2. Manuscript formatting

### 2.1. Case report

A 9-month-old neutered male Sphynx cat presented with hematochezia and chronic vomiting. The clinical signs began 5 weeks before the presentation, and the patient was administered a hypoallergenic diet based on a recommendation from a local animal hospital; however, the specific brand of the diet remained undisclosed. Despite this intervention, the patient continued to experience persistent diarrhea. Therefore, the cat was treated at a local hospital with amoxicillin with clavulanate, metoclopramide, prednisolone (PDS), and maropitant. Although the patient seemed to respond well to the therapy, but diarrhea recurred within 1 month.

The patient had no history of trauma or infection and was being administered anthelmintics and vaccination as recommended. The physical examination revealed normal body condition (body score of 4/9, 3.0 kg) and peripheral lymph nodes were palpably normal in size and texture. Neither respiratory signs nor evidence of dermatologic diseases was identified. A complete blood count using Procyte Dx (IDEXX Laboratories Inc., Westbrook, ME, United States) revealed a leukocytosis (19.40 × 10^9^/L; reference range, 2.87–17.02) with neutrophilia (15.97 × 10^9^/L; reference range, 1.48–10.29). Diagnostic tests performed included trypsin-like immunoreactivity (TLI) and feline pancreas-specific lipase (fPL) for pancreatic diseases, and SNAP Feline Triple kit (Feline SNAP Triple kit; IDEXX Laboratories Inc., Westbrook, ME, United States) for feline immunodeficiency virus, feline leukemia virus, and heartworm infection which were all negative. Diarrhea polymerase chain reaction (PCR) for Trichomonas fetus/blagburni, Cryptosporidium spp., Giardia spp., and Salmonella spp. all showed negative results, and no remarkable findings were identified on the fecal examination. Coagulation screening revealed shortened prothrombin time (PT) (13 s; reference range, 15–22) and activated partial thromboplastin time (aPTT) (62; reference range, 65–119), using Coag Dx (IDEXX Laboratories Inc., Westbrook, ME, United States). Abdominal ultrasonography was performed with an ultrasound device (IU 22; Philips Healthcare, Bothell, WA, USA) equipped with a linear transducer (L 12–5 5–12 MHz), and the patient was positioned in dorsal recumbency. On the abdominal ultrasonography, colitis and ileitis were considered, and mesenteric lymph node enlargement was identified. Fenbendazole (Panacur; Intervet Italia Srl) was administered at 125 mg/kg orally once to rule out parasitic diseases. The patient was once treated with cobalamin (250 mg/cat SC) (Actinamide inj.; Shinpoong Co., Seoul, Korea) for possible small intestinal diseases. Metronidazole (Flasinyl; CJ Healthcare Inc., Chengju, Korea) at 10 mg/kg BW orally (PO) every 12 h (q12h), tylosin (TearGuard, ELT Science Corp., Chengju, Korea) at 10 mg/kg PO q12h, and famotidine (Nelson Famotidine Tab, 20 mg; Nelson Corp., Chungcheongbuk-do, Korea) at 1 mg/kg PO q24h were prescribed for symptomatic treatment. The diet was changed to Hill’s Digestive Care i/d (Hill’s Pet Nutrition, Topeka, Kansas, United States).

**Figure 1:**
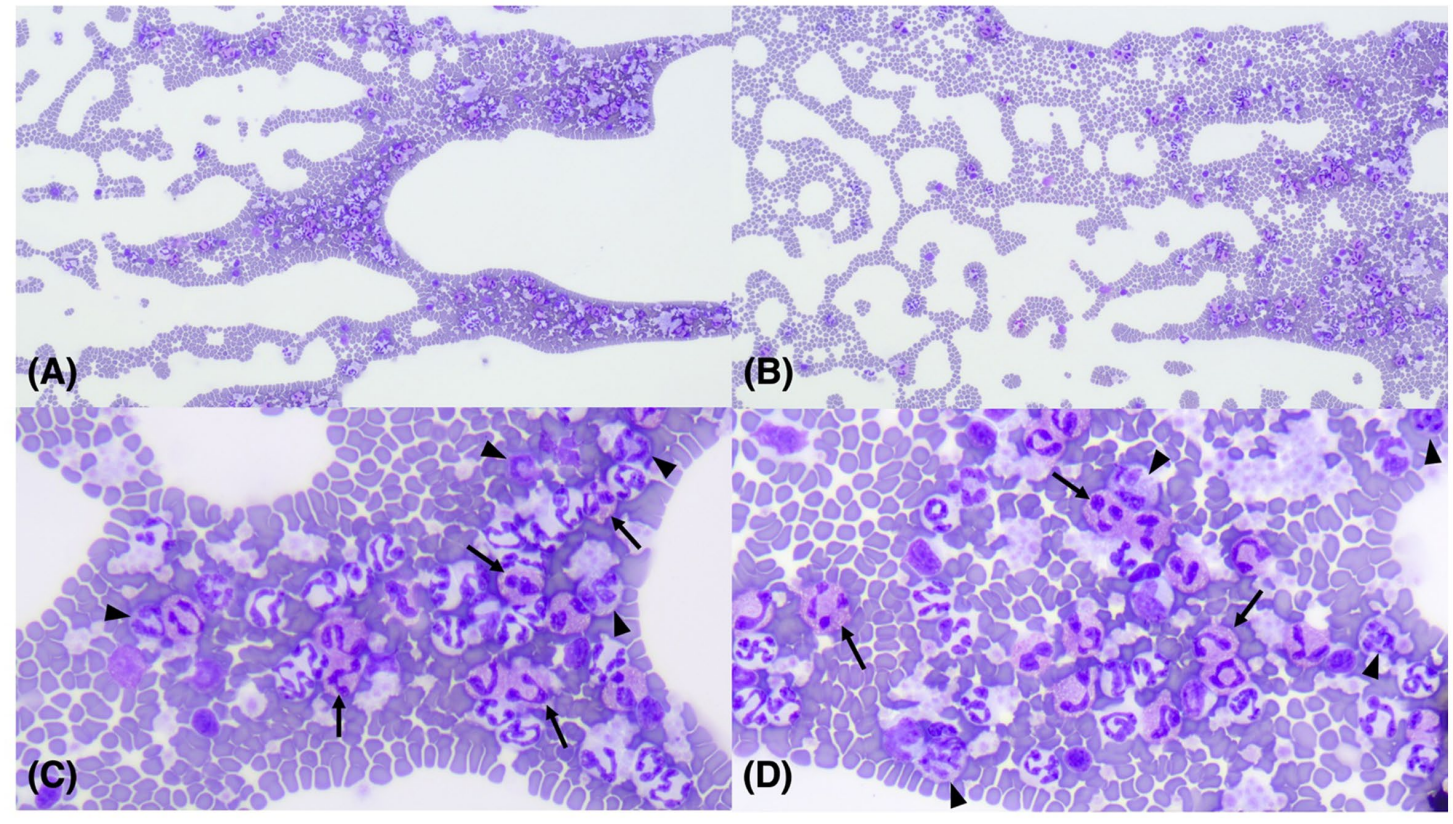
Images of microscopic examination of a 9-month-old, neutered, male Sphynx cat with basophilia and eosinophilia. Diff-Quik x 100 **(A,B)**; Diff-Quik x 400 **(C,D)**. Eosinophils (arrows) and basophils (arrowheads) were identified on the feathered edges of the blood smear.

**Figure 2:**
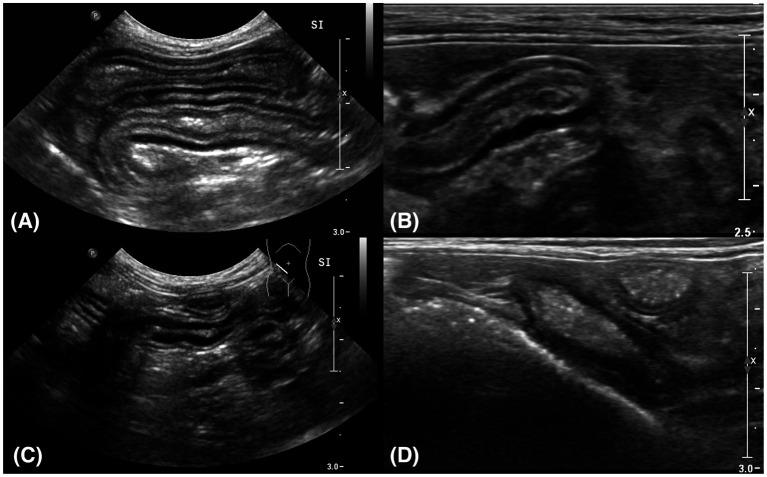
Sagittal plane ultrasound images of the small intestine **(A,C)** and ileum **(B,D). (A)** Thickening of the muscularis propria was identified in the small intestine. It was measured at 3.07 mm. **(B)** The thickened muscularis propria of the ileum was measured at 4.45 mm. **(C)** The thickness of the small intestine reduced to 2.37 mm after treatment. **(D)** The thickness of the muscularis propria of the ileum reduced to 2.36 mm after treatment.

**Figure 3:**
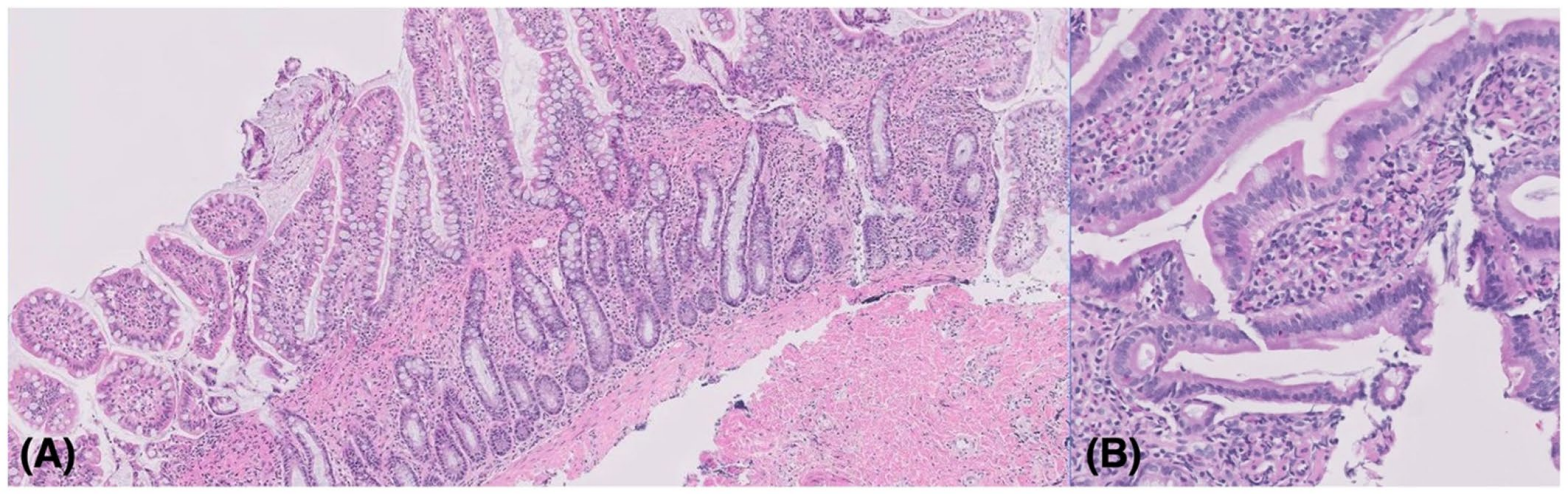
**(A)** Histopathology results from the surgical biopsy of the ileum. Mild to moderate diffuse lymphoplasmacytic and eosinophilic enterocolitis with collagenous enteropathy was identified. The lamina propria was mildly to moderately expanded by lymphocytes and plasma cells with multifocally mildly increased eosinophils. Hematoxylin and eosin stain, 100 x objective. **(B)** Histopathology results of the endoscopic biopsy. The findings were similar to the specimens obtained by the surgical biopsy. There was no overt histologic evidence of an infectious cause or neoplastic process. Hematoxylin and eosin stain, 200 x objective.

After the treatment, the clinical signs were alleviated, but the patient revisited the hospital 134 days after the initial presentation, with hematochezia as the chief complaint. Eosinophilia (2.50 × 10^9^/L; reference range, 0.17–1.60) and basophilia (0.79 × 10^9^/L; reference range, 0.01–0.30) were newly identified on manual differential counts (Figures 1A–D). Two days later, lymphocytosis (9.45 × 10^9^/L; reference range, 0.92–6.88) and monocytosis (1.84 × 10^9^/L; reference range, 0.05–0.67) were also identified on the complete blood count and manual differential count. Abdominal ultrasonography revealed an overall thickening of the muscularis propria in the small intestine (Figure 2A). The wall thickness of the muscularis propria of the ileum was 4.45 mm (Figure 2B). Some parts of the distal descending colon revealed an irregular mucosal layer. A hyperechogenic crater-like lesion was also identified on the colon, suggesting erosion or ulceration. Based on the ultrasound findings an endoscopy and colonoscopy were recommended to obtain biopsy samples. The biopsy samples for histopathology were obtained by the endoscopy (duodenum, cecum, colon) and surgical punch biopsy (ileum). On colonoscopy (EVIS EXERA III CLV-190; Olympus, Center Valley, PA, United States), there were no significant findings except the erosion of the cecum with some shallow craters. Multiple full-thickness sections of small intestinal mucosa obtained by the surgical punch biopsy during open abdominal surgery revealed mild to moderate expansion of the lamina propria by lymphocytes and plasma cells, along with multifocally mildly increased eosinophils (Figure 3A). No epithelial damage was noted, and no lesions were detected in the submucosa, muscularis, or serosa. A diffuse, moderate increase in the proprial sub-villous collagens with proliferating fibroblasts was identified. The endoscopic biopsy revealed a duodenum of which the lamina propria contained mildly to moderately increased numbers of eosinophils and appropriate to sometimes mildly increased numbers of plasma cells and lymphocytes (Figure 3B). Lamina propria of the colon contained mildly increased numbers of lymphocytes and plasma cells with low numbers of scattered eosinophils. There was no overt histopathologic evidence of infectious agents or neoplastic processes. Histopathologic lesions compatible with feline eosinophilic sclerosing fibroplasia were also absent.

**Figure 4:**
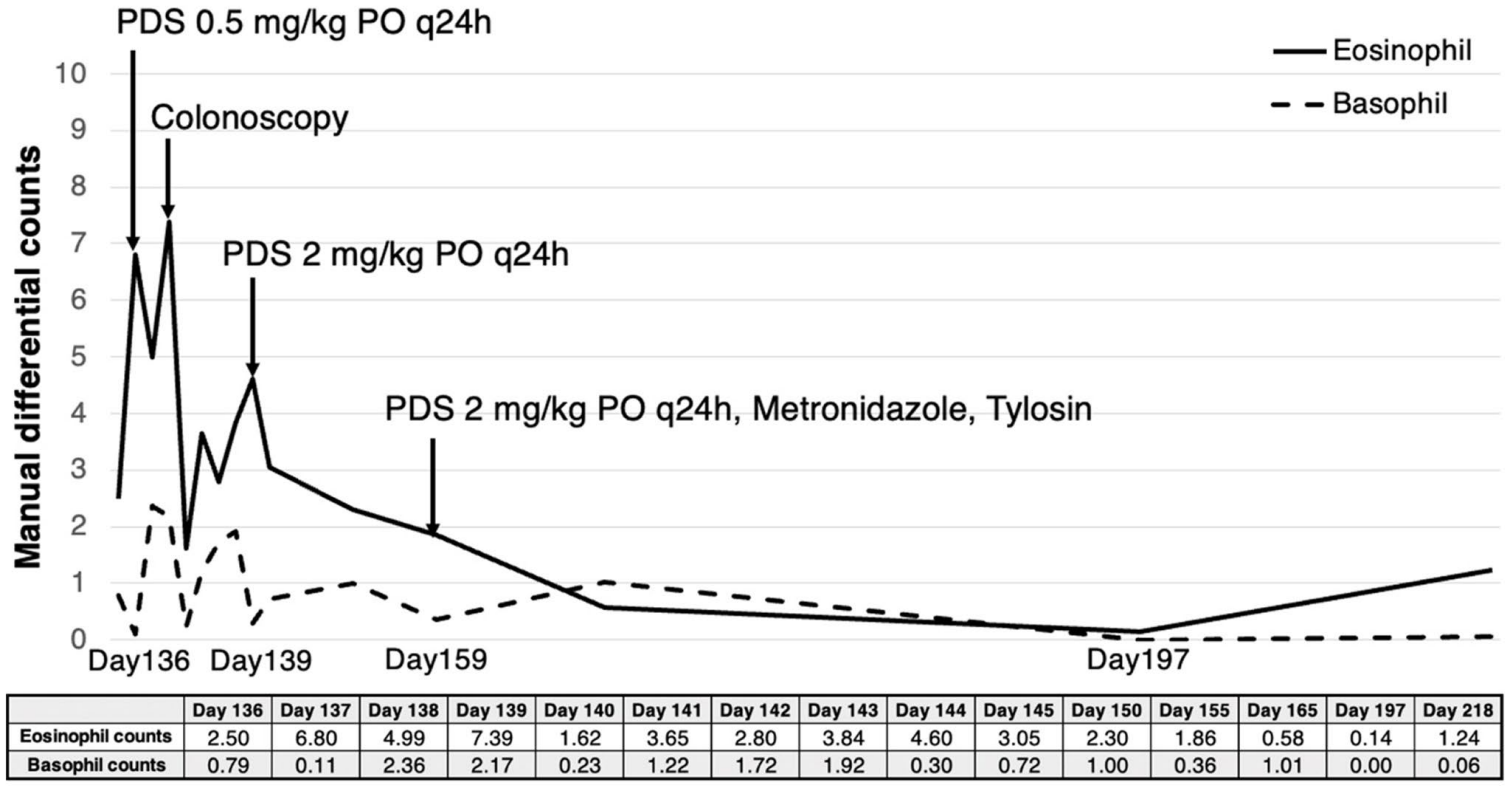
Eosinophil and basophil count measured by manual differential counting from the time when eosinophilia and basophilia were initially identified. After oral administration of PDS, the patient showed a gradual decrease in both eosinophil counts (reference range; 0.17–1.60 × 10^9^/L) and basophil counts (reference range; 0.01–0.30 × 10^9^/L). They returned to the reference range, and no further gastrointestinal signs were identified 8 weeks and 10 weeks after the initial treatment, respectively.

The patient was diagnosed with mild to moderate diffuse lymphoplasmacytic and eosinophilic enterocolitis, and the treatment was initiated with PDS (Solondo; Yuhan Inc., Seoul, Korea) at 2 mg/kg PO q24h, metronidazole (Flasinyl; CJ Healthcare Inc., Chengju, Korea) at 10 mg/kg PO q12h, and tylosin (TearGuard, ELT Science Corp., Chengju, Korea) at 10 mg/kg PO q12h. Eight weeks after the treatment started, the eosinophil and basophil counts returned to the normal reference range (Figure 4). Ten weeks after the treatment began, no further gastrointestinal signs were identified. On abdominal ultrasound, the thickening of muscularis propria of the small intestine, including the ileum, was alleviated (Figures 2C,D). Both tylosin and metronidazole were discontinued gradually, and PDS tapering began 11 weeks after the treatment began, starting from 2 mg/kg PO q24h to 1.5 mg/kg PO q24h. The dosage was tapered to 0.75 mg/kg PO q24h and 0.5 mg/kg PO q24h 18 weeks and 24 weeks after the treatment began, respectively. Neither recurrence of gastrointestinal symptoms nor eosinophilia with basophilia was identified during the 6-month follow-up period. Further PDS tapering is now still in process, at the time of writing.

### 2.2. Discussion

In veterinary medicine, basophilia has rarely been reported. Maria Balan et al. reported feline basophil prevalence for the first time in 2017, in which case the patient was diagnosed with alimentary T-cell lymphoma (2). Other reported feline basophilia cases included lymphoma and gastrointestinal eosinophilic sclerosing fibroplasia (FGESF) (8, 9). Thus, most feline veterinary cases with basophilia were related to tumor-associated paraneoplastic hypereosinophilia.

Various conditions can induce eosinophilia in cats. Hypersensitivity disorders, parasitism, lymphoma, eosinophilic leukemia, hypoadrenocorticism, and mast cell tumors can induce eosinophilia. In human medicine, a patient is diagnosed with idiopathic hypereosinophilic syndrome (HES) when blood eosinophilia (≥ 1.5 × 10^9^/L) is identified for longer than 6 months; when presumptive signs of organ involvement such as heart failure, gastrointestinal dysfunction, central nervous system abnormalities, fever or weight loss are identified; or when the evidence for parasitic, allergic, or other known causes of eosinophilia is lacking. If eosinophilia is limited to a single organ, such as in eosinophilic lung disease or eosinophilic gastroenteritis, it is excluded from HES (10). However, in veterinary medicine, there are no clear diagnostic criteria for HES, which is only diagnosed when all other causes of eosinophilia are excluded, with a rare incidence in cats (11–13). In the cat described in this study, neither respiratory signs nor dermatologic diseases were identified. All other causes, including pancreatic diseases, parasitism, and virus infection, were ruled out using TLI, fPL, fecal examination, and diarrhea PCR. However, in this case, HES was less likely to cause eosinophilia because lymphocytes and plasma cells were mainly identified on the biopsy, which was different from the pattern shown in HES. When HES is presented as gastrointestinal infiltration, marked eosinophilic infiltration alone is the major characteristic. As a result, the patient was diagnosed with lymphocytic-plasmacytic and eosinophilic enterocolitis, believed to be the cause of eosinophilia.

In human medicine, the effect of eosinophilia on the body system is well recognized. The infiltration of eosinophils in tissues can itself be pathologic if severe (14). Eosinophilia can promote fibroblast activation by secreting transforming growth factor-β and interleukin-1β and can induce further inflammation through an allergic mechanism (8). Most importantly, hypereosinophilia is a risk factor for venous thromboembolism (15, 16). An eosinophilic cationic protein released by eosinophils binds heparin and modulates thrombomodulin, resulting in an increased prothrombotic state. In our case, shortened PT and aPTT shown at the initial presentation were considered to have a low correlation with it since eosinophilia was not identified at that moment. When the patient revisited with eosinophilia and basophilia, clinical signs consistent with coagulation abnormalities or significant findings on physical examination and blood analysis were not identified to indicate coagulation analysis. However, if eosinophilia is consistently identified in cats in the future, it might be worthwhile to conduct routine coagulation tests as clinical vigilance for possible venous thromboembolism (17).

In veterinary medicine, establishing diagnostic criteria to differentiate feline IBD from low-grade intestinal T-cell lymphoma is an interesting subject. Differentiation is challenging since they share many similarities in signalment, clinical findings, lab tests, ultrasonographic findings, and cytology findings (7). However, as they show different outcomes and prevalence, differentiation is essential. So far, histopathologic diagnosis through biopsy has been considered a golden standard for differentiation (5, 7). In our case, physical examination, blood analysis, and ultrasonography had limitations in differentiating IBD from intestinal lymphoma. Thickened muscularis propria of the intestine was suggestive of IBD, but the overlap with lymphoma also exists (18). Feline alimentary lymphoma was initially suspected in the patient since the cat showed marked eosinophilia and basophilia, with the exclusion of parasitic causes, and had a fair response to treatment with glucocorticoid. However, the patient was finally diagnosed with lymphoplasmacytic and eosinophilic enterocolitis by histopathology, with no evidence of neoplasia and no specific histologic appearance consistent with feline FGESF. Thus, not only neoplasia but also enteritis should be considered in the diagnostic process if basophilia accompanied by eosinophilia is identified in cats in the future. Lymphocytosis identified in our case was supposed to be the manifestation of reactive lymphocytosis induced by nonspecific antigenic stimulation, considering the patient’s age. The presence of plasma cells with only mild lymphocytic infiltration on the biopsy specimens also suggested that lymphoma was a less plausible cause. However, chronic antigenic stimulation can recruit inflammatory T-cell lymphocytes with cytokines and inflammatory cells, leading to the clonal proliferation of lymphocytes (7). Thus, additional follow-up is required for the patient diagnosed with IBD.

The absence of a biopsy specimen at the jejunum, a commonly affected site in a low-grade intestinal T-cell lymphoma, is a possible limitation of our study. Also, there was no polymerase chain reaction for antigen rearrangement (PARR) information, but the possibility of a neoplasm was low, as identified by histopathology. Another possible limitation is the absence of cytokine analysis. Analysis of IL-5 and IL-3 may have enabled a more precise explanation of causality and underlying mechanisms.

### 2.3. Conclusion

This report describes clinical details, including manual differential counts, ultrasonography, endoscopy, histopathology, and the disease course of feline lymphocytic-plasmacytic and eosinophilic enterocolitis with marked basophilia and eosinophilia. Basophilia in cats is rare, and there have been few cases of feline basophilia accompanying eosinophilia reported in lymphocytic-plasmacytic and eosinophilic enterocolitis. Thus, in the future, if basophilia accompanied by eosinophilia is identified in cats, enteritis should also be considered as a possible cause. However, the effect of eosinophilia on cats has not yet been fully established and warrants further study. Furthermore, routine follow-up is required in patients with IBD considering its possibly progressive nature into feline alimentary T-cell lymphoma (7).

## Data availability statement

The original contributions presented in the study are included in the article/supplementary material, further inquiries can be directed to the corresponding author.

## Ethics statement

Ethical approval was not required for the studies involving animals in accordance with the local legislation and institutional requirements because since this is a single case report. Written informed consent was obtained from the owners for the participation of their animals in this study.

## Author contributions

J-YK performed clinical management of the case, wrote, and edited the manuscript. J-YK and J-HS contributed to the conception of the case report and revised the manuscript. T-SH, D-IJ, K-HS, and J-HS supervised the clinical management of the case. All authors contributed to the article and approved the submitted version.

## Funding

This research was supported by the National Research Foundation of Korea, funded by a grant from the Korean Government (NRF-2022R1G1A10036821131482092640101). This study is also supported by the Basic Science Research Program through the NRF, funded by the Ministry of Education (RS-2023-0021971031482092640001).

## Conflict of interest

The authors declare that the research was conducted in the absence of any commercial or financial relationships that could be construed as a potential conflict of interest.

## Publisher’s note

All claims expressed in this article are solely those of the authors and do not necessarily represent those of their affiliated organizations, or those of the publisher, the editors and the reviewers. Any product that may be evaluated in this article, or claim that may be made by its manufacturer, is not guaranteed or endorsed by the publisher.
